# Ultrasound quantification of tongue-palate distance and tongue thickness in TMD patients: changes after physical therapy and comparison with healthy controls

**DOI:** 10.1186/s12903-025-07568-w

**Published:** 2025-12-19

**Authors:** Yan Tang, Shi-Zhen Zhang, Ji-Ling Ye, Shen-Ji Lu, Yuan Yao, Zhong-Yi Fang, Xin Jiang

**Affiliations:** Department of Rehabilitation Medicine, Shanghai Ninth People’s Hospital, Shanghai Jiao Tong University School of Medicine, Shanghai, 200011 China

**Keywords:** TMD, Tongue-palate distance, Tongue thickness, Physical therapy, Ultrasound

## Abstract

**Background:**

Temporomandibular disorder (TMD) is closely linked to tongue function, yet the dynamic changes in resting tongue parameters (tongue-palate distance and tongue thickness) during TMD rehabilitation remain underexplored. This prospective, non-randomized controlled study aimed to quantify these parameters using ultrasound in TMD patients before and after physical therapy, and compare them with healthy controls, to clarify the therapeutic effect of physical therapy on tongue morphology.

**Methods:**

30 TMD patients and 30 healthy controls were enrolled. Ultrasound was used to measure tongue-palate distance and tongue thickness in controls once, and in TMD patients both before and after a standardized 2-week physical therapy protocol including thermotherapy, therapeutic ultrasound, transcutaneous electrical nerve stimulation (TENS), and mandibular exercises (3 sessions/week). The Mann-Whitney U test and Wilcoxon signed-rank test were used for between-group and within-group comparisons, respectively.

**Results:**

Results showed no significant change in tongue-palate distance in TMD patients post-treatment (P = 0.127), but a significant increase in tongue thickness (P < 0.05). Pre-treatment, TMD patients had significantly smaller tongue-palate distance (P < 0.05) and tongue thickness (P < 0.05) compared to controls; post-treatment, these parameters in TMD patients were comparable to controls (tongue-palate distance: P = 0.446; tongue thickness: P = 0.953).

**Conclusion:**

Physical therapy promotes tongue thickness change and brings tongue-palate distance closer to healthy levels, suggesting ultrasound-quantified tongue parameters could serve as objective indicators for TMD rehabilitation efficacy.

**Trial registry number:**

ChiCTR2000033328. Registration date: 2020-05-28.

## Background

Temporomandibular disorder (TMD) encompasses a spectrum of musculoskeletal conditions affecting the masticatory muscles, temporomandibular joint (TMJ), and associated structures, often leading to pain and functional impairment [1]. Emerging evidence underscores a significant interplay between tongue function and TMD pathophysiology. Specifically, altered tongue posture and function have been implicated in TMD pathogenesis, with studies reporting associations between unbalanced tongue position, abnormal swallowing patterns, and TMD symptoms [2, 3]. As a central component of the orofacial system, the tongue is integral to mastication, swallowing, and postural stability [4, 5]. Its functional capacity is governed by the complex interplay of intrinsic and extrinsic muscles, where muscle tone directly influences tongue morphology—hypertonia, for instance, may reduce tongue thickness by shortening muscle fibers [6]. Despite established clinical links, a critical knowledge gap persists regarding the role of resting tongue parameters, specifically tongue-palate distance and tongue thickness, in TMD. The resting tongue position serves as a biomechanical baseline for orofacial function, and deviations may reflect underlying muscular imbalances pertinent to TMD [4, 7].

Current methods for tongue assessment include lateral cephalometric radiography [8], computed tomography (CT) [9], magnetic resonance imaging (MRI) [10], three-dimensional ultrasound [11], and cone-beam computed tomography (CBCT) [12]. which are limited by cost, accessibility, and ionizing radiation, rendering them impractical for routine therapeutic monitoring.

Musculoskeletal ultrasound presents a compelling alternative, offering a non-invasive, real-time, and radiation-free modality for quantifying soft tissue morphology [13]. Its reliability for measuring tongue parameters has been established, with excellent intra- and inter-rater intraclass correlation coefficients (ICCs > 0.73) demonstrated in our study setup [14].

We posit that TMD-related muscular dysfunction is reflected in quantifiable alterations of resting tongue morphology. Furthermore, we base our intervention on the established capacity of physical therapy modalities (e.g., Low-level laser therapy, therapeutic ultrasound, TENS, specific exercises) to modulate muscle tone, perfusion, and function [15–18]. Building on this foundation, we hypothesize that: (1) Resting tongue-palate distance and tongue thickness differ significantly between TMD patients and healthy controls; (2) Rehabilitation treatment can normalize these parameters in TMD patients, bringing them closer to or aligning with those of healthy controls.

## Materials and methods

### General information

A priori power analysis (PASS v15.0.5) determined the sample size for detecting a clinically meaningful change in tongue thickness. Based on a paired t-test (α=0.05, two-sided; power = 0.90; effect size d = 0.67), 26 paired subjects were required. Accounting for potential non-normality and ~ 15% attrition, the target was set at 30 TMD patients and 30 matched healthy controls, ensuring robust detection of the therapeutic effect.

Thirty TMD patients (3 males and 27 females) who visited the outpatient department of the Shanghai Ninth People’s Hospital, Shanghai Jiao Tong University School of Medicine from March 1, 2023, to June 30, 2023, were recruited. Thirty healthy controls (4 males and 26 females) were recruited during the same period. The study was approved by the Medical Ethics Committee of the Ninth People’s Hospital Affiliated with Shanghai Jiao Tong University School of Medicine (ethics batch number: SH9H-2019-T316-2). Trial registry number: ChiCTR2000033328.

Healthy controls: The inclusion criteria were as follows: (1) good systemic health status; (2) no history of macrotrauma or surgical intervention involving the temporomandibular joint (TMJ) or cervical region; (3) no prior diagnosis of TMD; (4) absence of TMD-related signs and symptoms; no evidence of TMD upon brief clinical evaluation [19]; and (5) age should be 18 years old or older. Paticipants were excluded if they had the following characteristics: (1) with cognitive impairment, rendering them unable to cooperate effectively; or (2) those with known allergies to coupling gel.

TMD group: The inclusion criteria were as follows: (1) a diagnosis of TMD according to the DC/TMD [20]; (2) Only one side of TMJ is affected; and (3) patients aged > 18 years. Patients were excluded if they had the following characteristics: (1) a history of head or face trauma in the past 10 years; (2) systemic diseases, such as psoriatic arthritis, rheumatoid arthritis, gout, or other diseases affecting the masticatory system; (3) inflammatory, oncologic, or viral diseases of the face, or (4) those with known allergies to coupling gel.

All the participants were aware of the purpose of this study and signed an informed consent form. They were able to withdraw from the trial at any time.

The TMD patients included in this article are all patients with TMD symptoms who come for treatment. The diagnosis includes: degenerative lesions of the TMJ, TMJ disc displacement without reduction and no mouth - opening limitation, TMJ disc displacement without reduction and with mouth - opening limitation, muscle pain, joint pain, and TMJ disc displacement with reduction.

### Methods

#### Testing methods

Participants were seated comfortably on a standard-height chair (42 cm) without back support and were instructed to look straight ahead, maintaining their habitual head and neck posture.

##### Two evaluators participated in the measurement

**Fig. 1:**
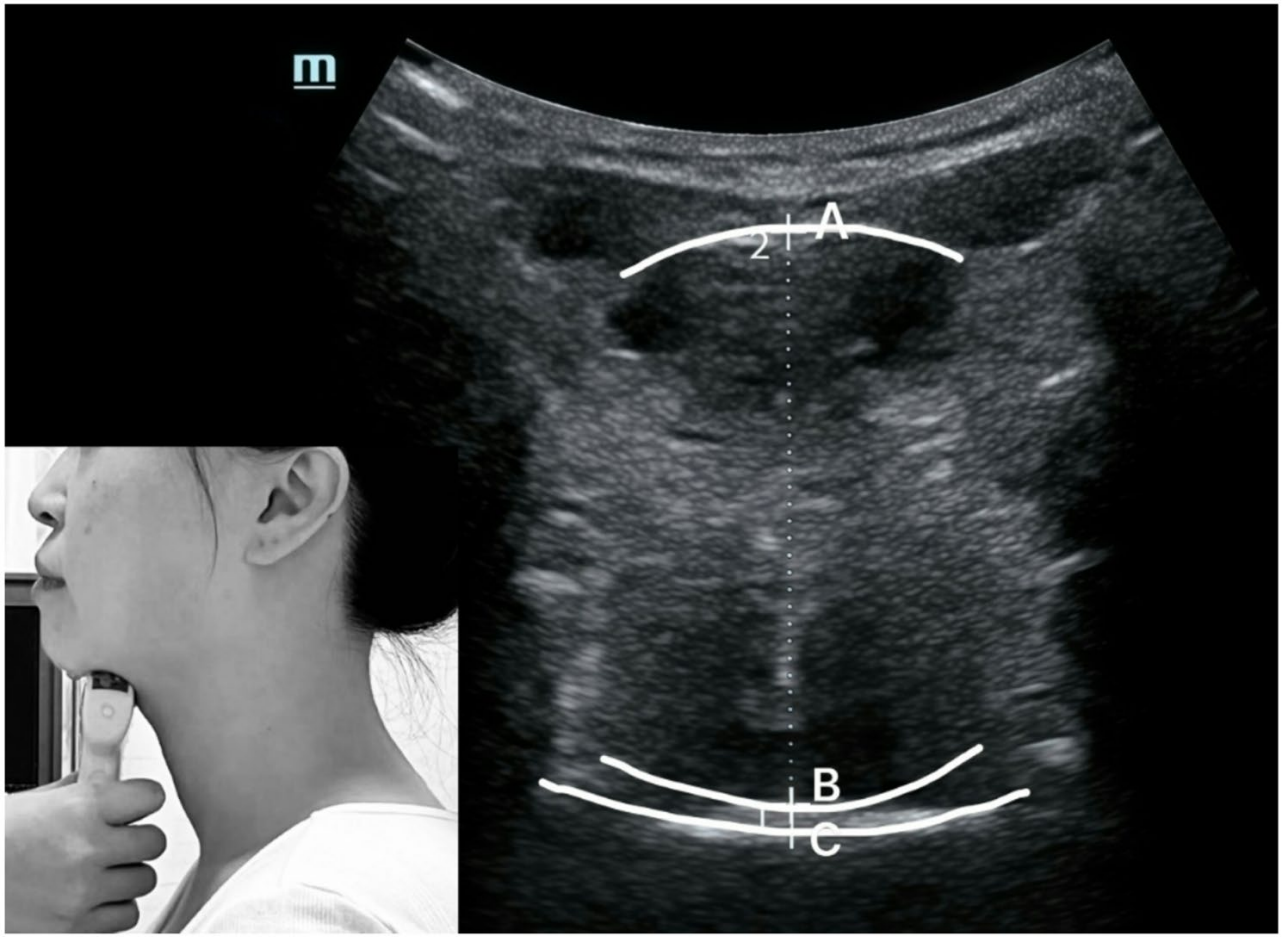
Ultrasound image scanned on the frontal plane of tongue. **A**: Basal lingual sole; **B**: Dorsal side of tongue; **C**: Surface of the palate. **AB**: Tongue thickness, **BC**: Tongue-palate distance

The first evaluator used Mindray ultrasound, UMT-500, and convex array probe C5-1s, Shenzhen Mindray Biomedical Electronics Co., Ltd. Initially, the probe was positioned at the anterior region of the subject’s mandibular floor, aligning with its long axis, before gradually moving towards the hyoid bone. The short axis was placed between the posterior aspect of the tongue and the upper palate, enabling the measurement and documentation of tongue-palate distance. Screenshots were captured to measure and record tongue thickness (Fig. 1). The ultrasound mode was set to a frequency of 5 MHz and a depth of 8 cm.

Tongue-palate distance: The distance between the tongue’s upper surface and the hard palate [21], in centimeters.

Tongue thickness: The vertical distance from the surface of the hyoid muscle to the back of the tongue [22], in centimeters.

**Fig. 2:**
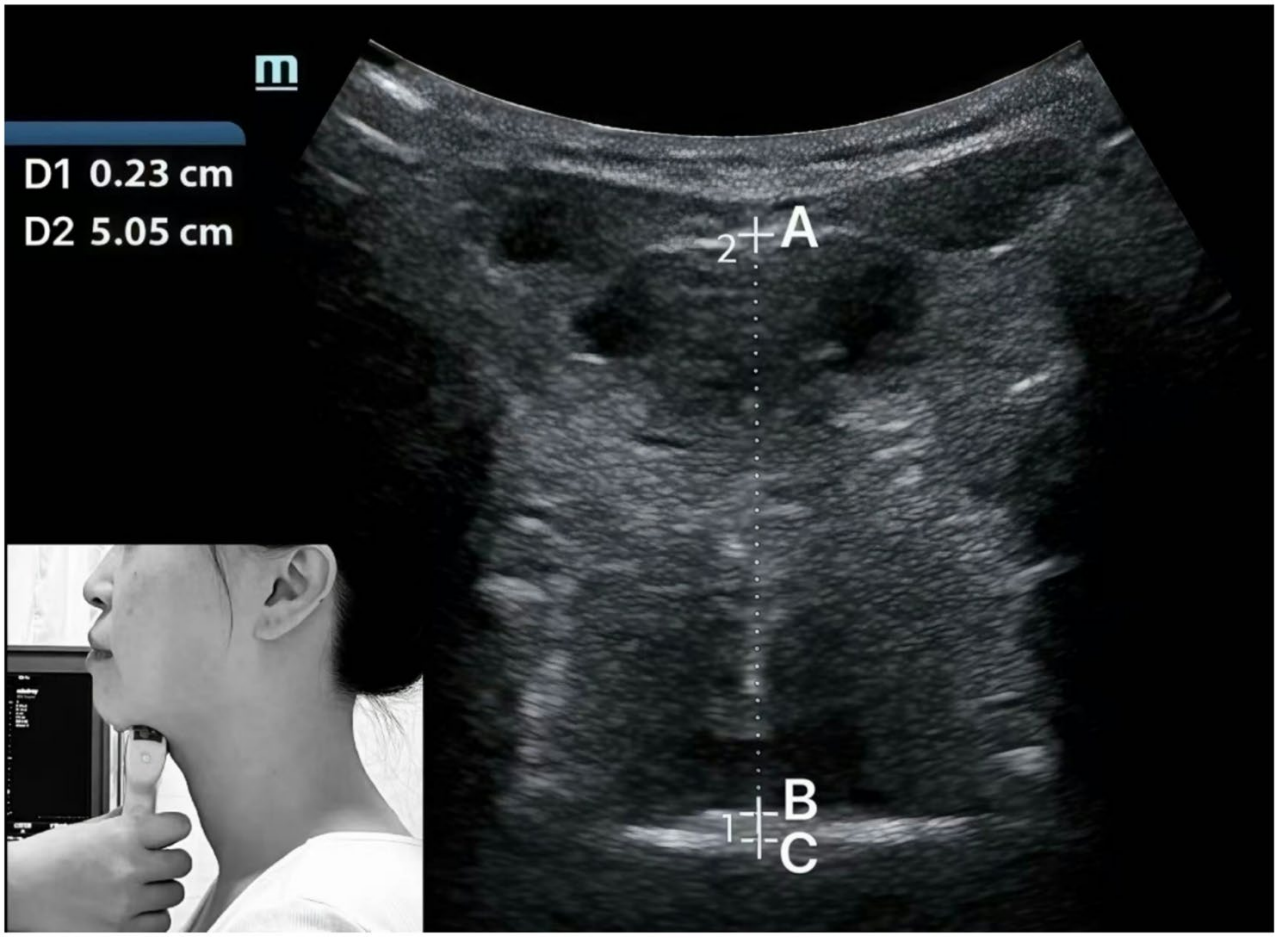
Measurements on the ultrasound image of tongue-palate distance and tongue thickness. **D1** = **BC**: Tongue-palate distance, **D2** = **AB**: Tongue thickness

The second evaluator independently reviewed the images saved by the first evaluator. Then measured the tongue-palate distance, as well as the tongue thickness, and recorded these measurements (Fig. 2).

The intra- and inter-evaluator intraclass correlation coefficients (ICCs) for tongue-palate distance are 0.745 and 0.611, respectively; for tongue thickness, they are 0.855 and 0.730. Individual evaluator reliability is consistent, with ICCs ranging from 0.761 to 0.788 for distance and 0.841 to 0.862 for thickness (all *p* < 0.05) [14].

The evaluators conducted only one measurement on healthy controls, while for TMD patients, measurements were carried out both before and after treatment.

### Rehabilitation protocol for TMD patients

All enrolled TMD patients completed the entire 2-week intervention protocol (6 sessions in total) with no dropouts. The treatment program included:

#### Health education [23]

Once a week, last for 10 min, including: (1) learning the normal resting position of the TMJ to rest the masticatory muscles; (2) observing and reducing parafunctional habits; (3) avoiding excessive mandibular movements, such as wide opening of the mouth; (4) maintaining a soft diet by cutting food into smaller pieces, and chewing carefully; (5) performing simultaneous bilateral mastication; and (6) improving posture.

#### Laser therapy

The patient takes a sitting position. A low-power semiconductor laser therapy device is used, with the probe attached to the most tender point of the masseter muscle. The treatment wavelength is 810 nm, continuous wave, power 60mW, once a day, 10 min per session for the unilateral side.

#### Ultrasound therapy

The patient takes a sitting position. An ultrasound therapy machine is applied, with a probe diameter of 3 cm. Coupling agent is applied to the TMJ and masseter muscle areas, using the contact movement method. The treatment frequency is 3 MHz, duty cycle 50%, 1.0 W/cm², once a day, 3 min per session for the unilateral side.

#### Transcutaneous Electrical Nerve Stimulation (TENs)

The patient takes a sitting position. A 2-electrode low-frequency therapy device is used, with electrodes placed at the most painful area reported by the patient during palpation. The phase duration is 50µs, the frequency is 100 Hz, and the intensity is set to the maximum level tolerable by the patient. The duration of each treatment is 10 min.

#### Shortwave therapy

The patient takes a sitting position. A shortwave therapy device is used, with both sides of the maxillofacial region placed between two electrodes with a spacing of 10 cm. The output power is 6–9 W/cm², the frequency is 27.12 MHz, and each treatment lasts 10 min, once a day.

#### Exercise treatment: including mobilizations, stretching, and manipulations of the TMJ

Health education was applied first, then the modality therapy, followed by exercise. None of the treatment therapists served as evaluators.

### Statistical analysis

This study employed a prospective, comparative observational design with pre- and post-intervention measurements in the TMD group, which was compared to a healthy control group recruited concurrently. Data analysis was performed using SPSS 22.0. Normality was assessed via the Shapiro-Wilk test. Normally distributed data were presented as mean ± SD and analyzed using independent t-tests, whereas non-normally distributed data were presented as median [IQR] and analyzed using nonparametric tests (Mann-Whitney U for two groups, Kruskal-Wallis with post-hoc tests for ≥ 3 groups, Wilcoxon signed-rank for paired data). Intergroup comparisons between TMD patients and controls used the Mann-Whitney U test. Statistical significance was set at two-tailed *P* < 0.05. To complement variability measures and enhance clinical interpretation, effect sizes (Cohen’s d for normally distributed data; r for non-parametric data) and 95% confidence intervals were calculated for all primary comparisons. Post-hoc power analysis was conducted using GPower 3.1 to determine the achieved power for detecting observed effect sizes, with α = 0.05 and the actual sample size (*n* = 30 per group).

## Results

### General information of subjects

Demographic and anthropometric characteristics of the participants, including age, body mass index (BMI), and diagnostic subtype of TMD group are summarized in Table 1. No significant difference in gender distribution was observed between groups (*P* > 0.05). Continuous data were expressed as mean ± standard deviation for normally distributed variables and median (interquartile range) for non-normally distributed variables. Nonparametric statistical analysis revealed no significant differences in baseline characteristics between the two groups (*P* > 0.05).

**Table 1:** General information of subjects

	Healthy controls	TMD Group
n	30	30
Male (n, %)	4, 13.33%	3, 10.00%
Female (n, %)	26, 86.67%	27, 90.00%
Age (years)	29.50(13.0)	28.50(12.5)
BMI(kg/㎡)	21.61 ± 2.75	19.86 ± 2.26
Diagnostic Subtype		
Myofascial Pain (n, %)		4, 13.33%
Arthralgia (n, %)		5, 16.67%
DDwR (n, %)		7, 23.33%
DDwRwIL (n, %)		0
DDwoR w/o Limitation (n, %)		11, 36.67%
DDwoR with Limitation (n, %)		2, 6.67%
Degenerative Joint Disease (n, %)		1, 3.33%

*DDwR Disc Displacement with Reduction, *DDwRwIL* Disc Displacement with Reduction with Intermittent Locking, *DDwoR w/o Limitation* Disc Displacement without Reduction, without Limitation, *DDwoR with Limitation* Disc Displacement without Reduction, with Limitation

### Tongue-palate distance of TMD patients and healthy controls before and after treatment

**Fig. 3:**
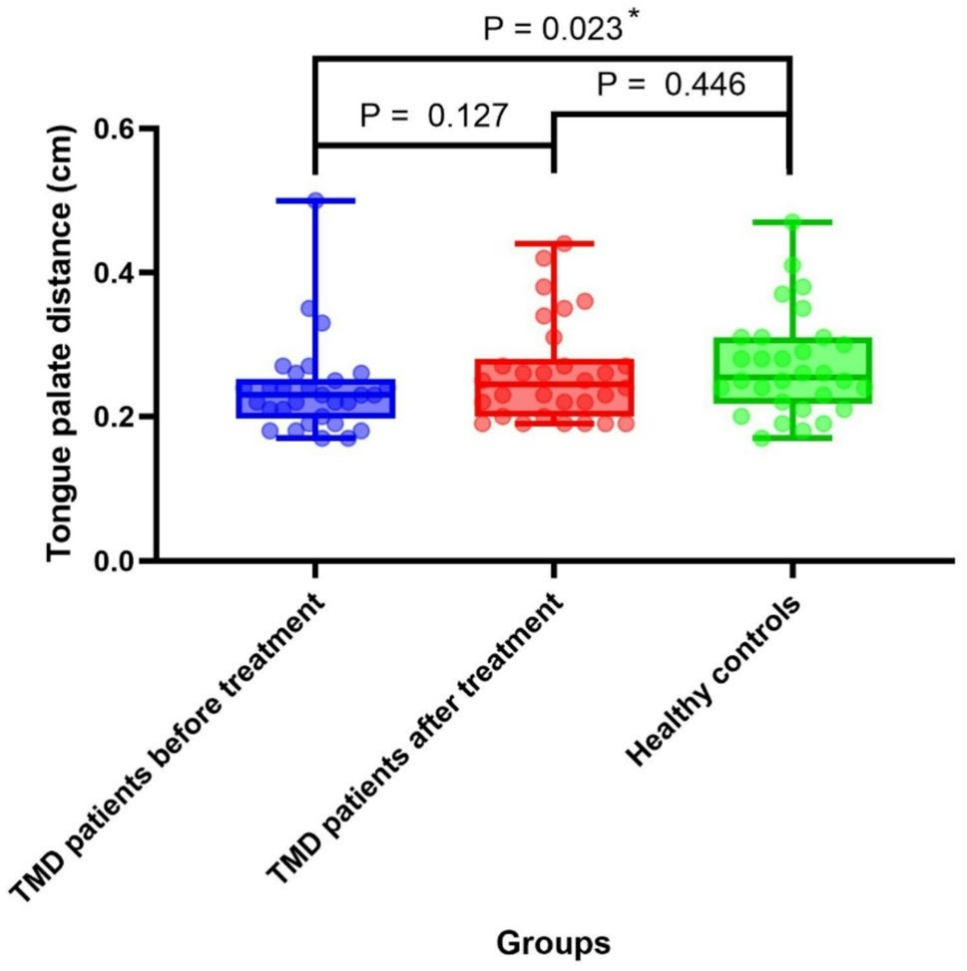
Tongue-palate distance of healthy controls and TMD patients (before and after treatment)

As illustrated in Fig. 3, the tongue-palate distance in TMD patients post-treatment (median[IQR]: 0.24 cm[0.08 cm]) showed a small effect size (*r* = 0.27, 95% CI [− 0.03, 0.05]) compared to pre-treatment values (0.23 cm[0.05 cm]), consistent with the non-significant difference (*P* = 0.127). Prior to treatment, the tongue-palate distance in TMD patients (0.23 cm [0.05 cm]) was significantly smaller than that in healthy controls (0.25 cm [0.09 cm]) (*P* < 0.05). Following treatment, the tongue-palate distance in TMD patients (0.24 cm [0.08 cm]) remained slightly smaller than that of healthy controls (0.25 cm [0.09 cm]), with no statistically significant difference observed between the two groups (*P* > 0.05). The post-hoc power for tongue-palate distance comparisons was 0.52–0.58 for detecting small effects (*r* = 0.27), indicating limited sensitivity that warrants cautious interpretation of non-significant findings.

### Tongue thickness of TMD patients before and after treatment compared with that of healthy controls

**Fig. 4:**
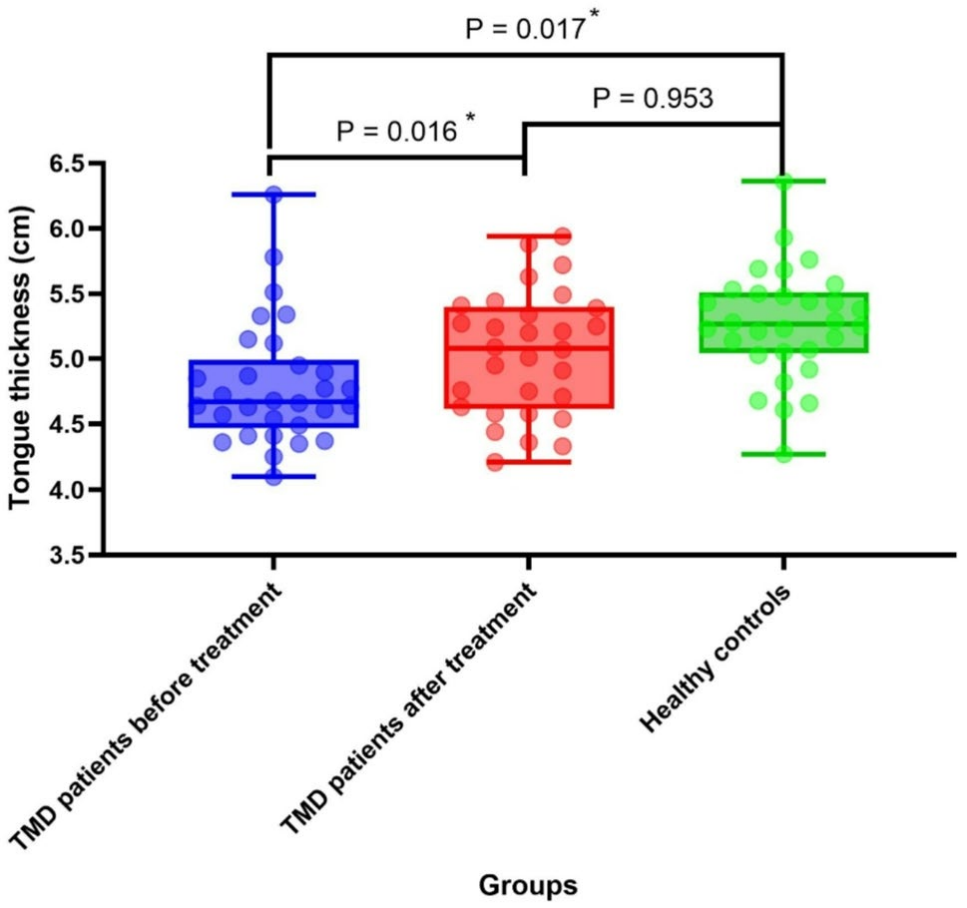
Tongue thickness of healthy controls and TMD patients (before and after treatment)

As illustrated in Fig. 4, tongue thickness increased significantly post-treatment (5.08 cm[0.78 cm] vs. pre-treatment 4.67 cm[0.52 cm], *P* < 0.05), with a medium effect size (Cohen’s d = 0.65, 95% CI [0.12, 0.80]), indicating clinically meaningful improvement. The significant increase in tongue thickness post-treatment may reflect reduced intrinsic muscle hypertonia6 and improved muscle perfusion (via ultrasound therapy), leading to normalized muscle fiber length and cross-sectional area. This aligns with Marim et al. [24], who reported increased tongue strength after TMD rehabilitation, supporting a link between muscle function and morphology. Prior to treatment, the tongue thickness of TMD patients (4.67 cm [0.52 cm]) was significantly smaller than that of healthy controls (5.06 cm [0.75 cm]) (*P* < 0.05). Following treatment, the tongue thickness of TMD patients (5.08 cm [0.78 cm]) exceeded that of healthy controls (5.06 cm [0.75 cm]), though this difference did not reach statistical significance (*P* > 0.05). For tongue thickness comparisons, the achieved power was 0.88–0.92, sufficient to detect medium effects (d = 0.65), confirming adequate sensitivity for this primary outcome.

## Discussion

This study utilized musculoskeletal ultrasound to quantify differences in tongue-palate distance and tongue thickness between TMD patients and healthy controls, both before and after rehabilitation. Our key findings revealed that following treatment, TMD patients exhibited an increase in tongue-palate distance (non-significant) and a significant increase in tongue thickness, with both parameters ultimately approaching the levels observed in healthy controls, which is consistent with the experimental hypothesis that rehabilitation would improve tongue morphology in TMD patients. These results provide novel insights into the role of tongue morphology and position in TMD pathophysiology and rehabilitation.

Our findings support the potential of ultrasound as an objective monitoring tool in TMD rehabilitation. However, randomized controlled trials with placebo groups are needed to confirm causal efficacy and establish clinical guidelines.

Impaired tongue function or reduced muscle strength can compromise masticatory and swallowing efficiency [24], and such functional deficits are closely linked to the onset or exacerbation of TMD [25]. However, the specific characteristics of tongue function in TMD patients remain poorly understood [26], with only limited evidence suggesting that chronic TMD may be associated with disrupted or weakened tongue function [24]. Our findings address this critical gap by demonstrating measurable changes in tongue parameters (thickness and resting position) following rehabilitation, highlighting that tongue morphology and position are dynamic traits amenable to intervention in TMD.

The tongue is a pivotal structure in orofacial function, contributing to speech, swallowing, and postural regulation [27, 28]. Its functional capacity is determined by multiple attributes, including size, strength, health status, movement patterns, and resting position [29]. The concept of “tongue rest position”-defined as the reproducible relaxed posture of the tongue when the lips are parted and the jaw is slightly open [30]-has long been recognized as a key marker of orofacial homeostasis. Nijdam and Teunissen [31] noted that a normal resting position typically involves the tongue tip contacting the upper alveolar ridge, while Wright and colleagues (cited in Baker [32]) observed that a “low lingual position” (tip below the mandibular incisor edge) is more prevalent than a “high position” (tip at the upper incisor or maxillary alveolar level). Consistent with these observations, our data showed that healthy controls had a greater tongue-palate distance (indicating a lower resting tongue position) compared to pre-treatment TMD patients. Following rehabilitation, TMD patients exhibited an increased tongue-palate distance, aligning their tongue position more closely with the common “low position” observed in healthy controls. The non-significant change in tongue-palate distance may be due to the short treatment duration (2 weeks); longer rehabilitation (e.g., 8 weeks) might be needed to alter habitual resting tongue position, as observed in myofunctional therapy studies [33]. Additionally, ultrasound may underestimate subtle anteroposterior tongue movements, which could affect tongue-palate distance assessment [26]. This finding aligns with a prior report suggesting that systemic rehabilitation may improve tongue position in TMD patients, albeit not significantly—a result potentially due to the subjective nature of patient-reported assessments in that study [33]. Our objective ultrasound measurements extend this work by quantifying these positional changes, supporting the utility of objective assessments in tracking therapeutic effects.

The tongue is integral to the jaw’s sensorimotor system: in the absence of visual cues, young healthy adults rely on tongue pressure against the hard palate (behind the teeth) to detect sensory inputs and adapt to environmental changes, thereby enhancing postural control by reducing center of gravity (COG) velocity [4]. This mechanism may be relevant to TMD, as myogenic TMD patients (without spontaneous pain) exhibit greater postural instability-characterized by increased sway area and velocity-compared to healthy controls [34]. If this sensorimotor mechanism is impaired in TMD, one might expect alterations in resting tongue posture as part of the compensatory adaptation. Our findings are consistent with this notion, as pre-treatment TMD patients had a smaller tongue-palate distance, which may reflect increased tongue pressure against the hard palate as a compensatory strategy to stabilize posture. Post-treatment, the increased tongue-palate distance suggests reduced palatal pressure, potentially aligning with improved postural control. However, the direct correlation between tongue-palate distance and postural stability warrants further investigation.

The tongue is a complex skeletal muscle structure, composed of interwoven intrinsic and extrinsic muscles with distinct directionalities [7, 35]. Intrinsic muscles (longitudinalis, verticalis, transversus) modify tongue shape (shortening, thinning, narrowing, respectively) [36], while extrinsic muscles govern gross tongue movement [35]. Balanced tension among these muscles is critical; imbalance can disrupt hyoid position and tongue function [2]. Muscle tone-sustained tension in relaxed muscles due to involuntary motor unit activation [6]-may play a role here: our finding of significantly smaller pre-treatment tongue thickness in TMD patients (compared to healthy controls) could reflect increased intrinsic muscle tone, leading to tongue thinning. This aligns with observations in masseter muscles, where TMD patients exhibit reduced thickness (18.8% in relaxed state, 15.9% in contracted state) compared to non-TMD individuals [37]. Notably, post-treatment increases in tongue thickness (approaching healthy levels) suggest that rehabilitation alleviates intrinsic muscle hypertonia, restoring normal tongue morphology.

### Limitations

This study has several limitations. First, the relatively small sample size, while adequate for detecting medium effects (e.g., in tongue thickness), may have limited power for identifying subtle changes (e.g., in tongue-palate distance). Furthermore, the short-term (2-week) intervention and lack of long-term follow-up preclude assessments of effect durability. Second, the potential for measurement bias exists, as evaluators were not blinded to group allocation or assessment time points, despite standardized protocols to minimize this risk. Third, the study design—specifically, the multifaceted intervention without a sham control and the lack of subgroup analysis by TMD diagnosis—limits our ability to isolate the effects of specific therapeutic components or to explore subtype-specific responses.

### Future directions

Subsequent studies will classify TMD patients by diagnostic subtype to explore whether tongue parameters (position, thickness) differ across subgroups. Additionally, integrating tongue assessments with postural stability and masticatory efficiency measures may clarify the mechanistic links between tongue function and TMD pathophysiology. Future research could also evaluate tongue morphology in patients with different types of malocclusion before and after orthodontic treatment.

## Conclusion

Musculoskeletal ultrasound showed that pre-treatment TMD patients had significantly different tongue-palate distance and tongue thickness from healthy controls. After systematic rehabilitation, notable changes occurred: tongue thickness increased significantly vs. pre-treatment, and both parameters no longer differed significantly from healthy controls.

These findings indicate that the rehabilitation program is associated with measurable changes in tongue morphology and resting position among TMD patients - specifically, an increase in tongue-palate distance and tongue thickness—bringing these parameters closer to healthy norms. Thus, ultrasound quantification of these parameters may serve as valuable indicators for evaluating treatment responses and guiding personalized TMD rehabilitation. 

## Data Availability

Study data are clinical patient data that cannot be made openly available. For inquiries about the data and collaborations please contact the corresponding author.

## Abbreviations

TMD: Temporomandibular disorder
TMJ: Temporomandibular joint
ICC: Intraclass correlation coefficient
CT: Computed tomography
MRI: Magnetic resonance imaging
CBCT: Cone-beam computed tomography
Me: Median
IQR: Interquartile range
BMI: Body mass index
DDwR: Disc Displacement with Reduction
DDwRwIL: Disc Displacement with Reduction with Intermittent Locking
DDwoR w/o Limitation: Disc Displacement without Reduction, without Limitation
DDwoR with Limitation: Disc Displacement without Reduction, with Limitation
COG: Center of gravity
